# Implementing the Patient Needs in Asthma Treatment (NEAT) questionnaire in routine care: a qualitative study among patients and health professionals

**DOI:** 10.1186/s12890-022-02293-4

**Published:** 2023-01-17

**Authors:** Julia Salandi, Patricia Vu-Eickmann, Christian Apfelbacher, Aziz Sheikh, Adrian Loerbroks

**Affiliations:** 1 Institute of Occupational, Social and Environmental Medicine, Centre for Health and Society, Faculty of Medicine, Heinrich-Heine-University Duesseldorf, Moorenstr. 5, 40225 Duesseldorf, Germany; 2 Institute of Social Medicine and Health Systems Research (ISMHSR), Medical Faculty, Otto von Guericke University Magdeburg, Leipziger Str. 44, 39120 Magdeburg, Germany; 3 Asthma UK Centre for Applied Research, Usher Institute, Old Medical School, University of Edinburgh, Teviot Place, Edinburgh, EH8 9AG UK

**Keywords:** Asthma, Disease management, General practice, Needs, Patient-reported outcome measures, Qualitative study, Pulmonary rehabilitation, Therapeutics

## Abstract

**Background:**

Many patients with asthma report unmet health care needs. The *Patient Needs in Asthma Treatment* (NEAT) questionnaire is a validated instrument to quantify these unmet needs. We explored how health professionals evaluated the instrument’s utility as well as patients’ and professionals’ perspectives of how NEAT could be incorporated into routine clinical practice.

**Methods:**

Qualitative interviews were conducted by telephone between February and September 2021 with 19 patients with asthma and 21 health professionals (i.e., general practitioners, pneumologists, health professionals in pulmonary rehabilitation, and medical assistants). Interview recordings were transcribed verbatim and content-analyzed using both deductive and inductive approaches using MAXQDA.

**Results:**

Health professionals could see the potential value of using NEAT to inform clinical decisions. However, health professionals tended to be skeptical towards the routine use of NEAT in outpatient settings, mainly due to a lack of time. Implementation of NEAT was seen as more valuable in the context of patient education (i.e., in Disease Management Programs [DMPs] or pulmonary rehabilitation) by patients and health professionals alike, because it offered greater opportunities to address any unmet needs identified. Both patients and health professionals considered it more useful to use the questionnaire for the first time some time after the initial diagnosis has been made (e.g., when the treatment regime is found rather than at time of initial diagnosis). In the context of DMPs and pulmonary rehabilitation, NEAT could be used twice, i.e., before and after patient education to support patient-centered planning and evaluation.

**Conclusion:**

Both patients and health professionals consider the use of the NEAT, in particular in educational programs (i.e., during DMPs or pulmonary rehabilitation), as feasible and useful. There is now a need to undertake a feasibility trial in routine care.

**Supplementary Information:**

The online version contains supplementary material available at 10.1186/s12890-022-02293-4.

## Background

Patients with asthma frequently report unmet needs in the context of their treatment (e.g., for more information) and seem to perceive and evaluate aspects of their treatment (e.g., the quality of communication or the time spent on disease education) differently than the treating physicians [1, 2]. Addressing unmet health care needs through individualized treatment approaches may facilitate physician-patient communication and help improve adherence and asthma outcomes (e.g., better asthma control) [1]. However, an essential prerequisite for this is that health care needs can be measured in a reliable and valid fashion. Since no instrument existed to measure such needs in asthma, we developed the *Patient Needs in Asthma Treatment* (NEAT) questionnaire with strong patient involvement [3]. We examined its psychometric properties cross-sectionally [4, 5] and longitudinally [5]. That work demonstrated that the NEAT is a reliable and valid instrument to identify unmet health care needs. We found that it is responsive to changes in treatment satisfaction and in asthma-related quality of life [5]. After its validation, the NEAT was administered in three rehabilitation clinics and we were able to provide preliminary evidence that pulmonary rehabilitation in adults with asthma may reduce asthma-related health care needs [6].

In terms of clinical decision-making, the NEAT could possibly facilitate the delivery of patient-centered care, which includes the tailoring of treatment to patients’ needs [7]. Thus, health care needs, measured by the NEAT, could represent an important additional patient reported outcome measure (PROM) to, for example, improve asthma control and asthma-related quality of life [8]. In particular, the tool could prove helpful in understanding health care needs at the time of diagnosis and throughout the further course of the disease (e.g., in case of poor asthma control) [5].

This is based on the premise that health professionals would find the instrument acceptable, useful and its implementation feasible in routine clinical practice. Insights into the (dis)similarities between patients’ and health professionals’ views have the potential to allow for implementation approaches that are patient-centered, but also pragmatic and feasible according to the health professionals. We therefore explored how health professionals evaluated the instrument’s utility and how patients and health professionals would incorporate the NEAT into routine clinical practice.

## Methods

### Study design

To address the above-mentioned research questions, we undertook a qualitative study using telephone-based semi-structured interviews for data collection.

### Development of the topic guides

We developed separate topic guides for patients and health professionals prior to data collection (see Additional file 1 and 2). In order to be able to explore the research questions, we included questions on the utility of the NEAT (e.g., “What do you think about the use of the questionnaire in your daily work?”) and potential implementation modes (e.g., “How, when, where and by whom should the tool be applied?). The topic guides were refined throughout data collection in response to the emerging insights [9]. After the first five interviews, feedback was given to the interviewer (i.e., JS) by AL, who is experienced in qualitative health research with patients with asthma [10, 11] and health professionals [12, 13]. Interview techniques were discussed and potentially refined. Certain questions were rephrased for better understanding, and open-ended opening questions were introduced as an icebreaker (for patients: “Perhaps you could start by telling me how satisfied you are with your current asthma treatment?”; for health professionals: “Could you start by briefly telling me how you work with patients with asthma in your day-to-day work?”). This feedback also made it possible to address *reflexivity*, i.e., the subject influence of the interviewer during the research process. After approximately half of the interviews were conducted, further adjustments took place, and a more multi-perspective interview technique was chosen. For example, health professionals were specifically asked about views and suggestions by other interviewed health professionals (e.g., which types of health professionals should administer the NEAT to patients?).

### Measures

#### Sample 1: Patients

To obtain supplementary information, patients were asked to complete a short standardized online survey prior to the interview to collect demographic and health-related background data, including the domains as follows.

**Patients’ health care needs** To familiarize patients with NEAT, they were asked to complete the questionnaire during the online survey. The 13-item questionnaire measured needs on four subscales, i.e., consideration of patient expertise by physicians (4 items); information on drug effects (3 items); information and training related to handling of drugs (3 items); responding to exacerbations (3 items). Items were phrased as questions with three response options: “Yes, I would like this”, “This need has already been met”; and “No, I do not need this” [3].

**Perception of disease** Furthermore, we used the following three items from the Brief Illness Perception Questionnaire (IPQ) to obtain a brief self-assessment from patients on how they were doing with their disease and treatment at the time of the interview: (a) “How much do you experience symptoms from your illness?, (b) “How much control do you feel you have over your illness?”, and (c) “How much do you think your treatment can help your illness?”. The Brief IPQ is a nine-item scale designed to assess the patients’ cognitive and emotional representations of illness [14]. Numeric rating scale with anchors at “0 = e.g., no symptoms at all” and “10 = e.g., many severe symptoms” are used [14]. The items chosen here have not been validated for separate use, but were deemed useful by the study team to briefly assess illness perceptions of the participants.

**Demographics** In order to describe the patient sample appropriately, we collected data on sex, age, years since asthma diagnosis and school education. In addition, we asked for prior experience with the NEAT tool (e.g., during previous surveys).

#### Sample 2: Health professionals

To collect demographic and occupational data from the health professionals we asked a few questions during the interview (e.g., type of profession and working experience in patient care in years).

### Participant recruitment

Between February and September 2021, we conducted 19 qualitative interviews with patients who reported physician-diagnosed asthma and 21 qualitative interviews with health professionals, these were general practitioners, pneumologists, health professionals in pulmonary rehabilitation, and medical assistants.^1^

#### Sample 1: Patients

Patients with asthma were recruited through an internal registry, i.e., patients who had previously participated in other asthma care studies with our research team and had given written consent to be re-contacted within the next 10 years. A total of 150 patients were contacted by e-mail. Eight e-mails could not be delivered and four patients gave feedback that they did not want to participate in further studies (due to data security concerns or because there experienced no symptoms at all). A total of 31 patients (20.7%) completed the online questionnaire and 21 of them (14.0%) agreed to a telephone interview. Finally, telephone interviews could be conducted with 19 patients; we thus achieved a response rate of 12.7%. Patients were at home during the interview.

#### Sample 2: Health professionals

We stratified the recruitment of health professionals by initially three subsamples of clinicians involved in different domains of asthma care; these were (a) general practitioners (GPs, primary care), (b) pneumologists in secondary care, and (c) health professionals in pulmonary rehabilitation involved in the care and/or education of rehabilitants with asthma (i.e., physicians, physiotherapists and physician assistants^2^). We recruited pneumologists and GPs by using an internal registry that included physicians who had previously participated in one of our previous asthma studies [5]. Those physicians received an invitation to participate as well as the study information by post (GPs: n = 25, pneumologists: n = 46). Furthermore, with the support of the “HausarztNetz Düsseldorf” (HAND e.V., a network of GPs) we were able to reach another 144 GPs via an e-mail list. We were able to recruit the pulmonary rehabilitation staff with the help of two pulmonary rehabilitation clinics that had already supported us in previous research studies [6, 10, 15–17] (the number of potential participants contacted is unknown). After initial analyses, we decided to additionally survey medical assistants in pneumological or general practices given that Disease Management Programs^3^ (DMPs) are also offered in the respective practice and the medical assistant confirms to have regular contact with patients with asthma in her/his daily work. We decided to include medical assistants as our interviews with health professionals and patients suggested that medical assistants may play a crucial role in meeting various health care needs queried in the NEAT (e.g., regarding the correct use of asthma inhalers). Furthermore, it became clear that physicians in outpatient settings were perceived (by others and themselves) to be unable to explore and specifically consider all health care needs themselves due to time constraints. Medical assistants were contacted with the help of the German Association of Medical Professions (“Verband medizinischer Fachberufe e.V.”) by social network post and personal requests.

In total, 5 GPs (response rate: 3.0%), 5 pneumologists (response rate: 10.9%) 7 healthcare professionals in pulmonary rehabilitation (3 pneumologists, 2 physiotherapists and 2 physician assistants, response rate unknown) and 4 MAs (response rate unknown) participated. Overall, telephone interviews were conducted with 21 health professionals who were either at work or at home during the interview.

### Data collection and analysis

All interviews were conducted by JS (M.Sc., female), who has a background in psychology and pulmonary health services research [5, 6, 18]. Interviews were conducted until thematic saturation was reached, i.e., if it could be assumed that the inclusion of further individual cases does not result in further topic areas or greater knowledge [9]. Interviews were digitally recorded, transcribed and content-analyzed using the software package MAXQDA. Qualitative content analysis was performed according to Mayring and using an iterative analytical approach involving multiple analysts [19]. Main categories were formed deductively according to the interview guides. In the first analysis round, individual statements were systematically sorted and grouped into the main categories (coding). In this way, subcategories (i.e., based on the findings from the interviews) were formed inductively. Initially, two different analysts (JS and PVE [doctoral degree, female]) coded four patient and four health professional interviews in this manner. Subsequently, the two preliminary coding schemes were compared and the coding of the individual statements was partially reordered. In this way, the subcategories could be refined and supplemented. JS then used these initial coding schemes to analyze all transcripts accordingly, adding additional subcategories as necessary. In order to deepen our analyses, a second round of coding was performed by JS. The interviews and coding of data were then reviewed by and discussed with PVE and AL [professor, doctoral degree, male] and the coding structure was slightly modified mainly with regard to *replicability* (i.e., formation and designation of categories and coding of individual statements), *traceability* (Does the coding scheme seem logical?), and *discriminatory power* (Is there content overlap between various categories?). This led to the final coding scheme, which was then again applied to all transcripts in a third round of coding (JS). Subsequently small modifications were discussed conclusively until consensus was reached (AL and JS). Finally, we created separate coding scheme for patients and health professionals. However, both coding schemes displayed a high degree of structural consistency to better compare the results. Additional file 3 presents the completed checklist of consolidated criteria for reporting qualitative research (COREQ) [20] to thereby further increase *transferability*.

## Results

### Demographics

In total, we interviewed 19 patients who reported physician-diagnosed asthma. The interview duration was 18.8 min on average (range 11.5–30.0 min). More women than men were interviewed (73.7%). Participants were, on average, in their mid-fifties and had mostly attained high levels of school education. They reported an average of 3.37 unmet health care needs, as measured by the NEAT (potential range 0–13). Regarding the disease perception questions (from the Brief IPQ), participants indicated the following: they currently experienced moderate symptoms caused by their asthma (i.e., a mean score of 4.89 out of 10), but reported high levels of asthma control (8.74 out of 10) and were convinced that their treatment could be highly helpful for their asthma (9.63 out of 10). Eight of the 19 participants reported having completed the NEAT in a previous study. Characteristics of the patient sample can be found in Table 1.

**Table 1:** Characteristics of the patient sample

Variables	Sample 1—Patients group (n = 19)
Female sex, *n*	14
Age (years), mean (SD)	54.12 (8.23)
Years since asthma diagnosis, mean (SD)	20.26 (11.15)
School education, *n*	
Low	4
Middle	3
High	12
NEAT total score, mean (SD)	3.37 (2.77)
IPQ item “symptoms”, mean (SD)	4.89 (1.82)
IPQ item “asthma control”, mean (SD)	8.74 (0.81)
IPQ item “help through treatment”, mean (SD)	9.63 (0.96)
Prior experience with the NEAT (yes), *n*	8

In total, we interviewed 21 health professionals (5 GPs, 5 outpatient pneumologists, 4 outpatient medical assistants and 7 members of pulmonary rehabilitation staff). The interview duration was 25.0 min on average (range 15.4–39.3 min). About half of the participants were female, and the average work experience in health care was 22.9 years. The vast majority of health professionals found the NEAT very useful for patient care (for general practice: 16/21, for pneumological practice: 17/21, for pulmonary rehabilitation: 21/21). Characteristics of the clinician sample can be found in Table 2.

**Table 2:** Characteristics of the clinician sample

Variables	Sample 2—Health professionals (n = 21)
Profession, *n*	
General practitioners (GPs)	5
Outpatient pneumologists	5
Outpatient medical assistants	4
Pulmonary rehabilitation staff	7
Pneumologists	3
Physiotherapists	2
Physician Assistants	2
Female sex, *n*	11
Years in patient care, mean (SD)	22.89 (9.95)
“NEAT is very useful for general practice.” *n* ^1^	16
“NEAT is very useful for pneumological practice.” *n* ^1^	17
“NEAT is very useful for pulmonary rehabilitation.” *n* ^1^	21

^1^Shown are all those who chose one of the last two options for the response options “do not agree at all,“ “do not agree,“ “agree,“ “fully agree”

### Results of qualitative interviews

All 19 patient interviews and 21 interviews with health professionals were included in the qualitative content analysis. The topics that were relevant to the exploration of the research question are detailed below (i.e., utility from clinicians’ point of view [overall impression, physician-patient communication] and implementation modes [setting, time of first deployment, frequency, responsible contact person, barriers]). In order to limit the scope of the content, not all topics of the coding scheme could be considered. For example, suggestions regarding the wording of individual items were not included, because  the NEAT is already a patient-centered and validated instrument [3]. Statements illustrating each category are provided as verbatim quotes in Table 3.

**Table 3:** Verbatim quotes, listed according to categories of the coding scheme

Topic		Verbatim quotes	Participant
*Utility from clinicians’ point of view*			
Overall impression	Q1	You really managed to write down all important questions an asthmatic could possibly have. I don’t think that anything is missing. I’ve read the questionnaire a couple of times and I can’t think of anything that should be added straightaway.	Medical assistant, pulmonary rehabilitation (female, 30 years in patient care)
Physician-patient-communication	Q2	Well, there’s something positive about it. You can take this questionnaire and you can give it to the patients in the waiting room and then you can have a glance at it, and they can tell you: Yes, I would like to have this or that. And this is really helpful for us as physicians because we know that we have to pay special attention to certain aspects.	General practitioner (male, 32 years in patient care)
	Q3	What I really like is that you can use the questionnaire to address special issues in weekly ward rounds and you can effectively address the patient and go into more depth there, or you can adapt training programs accordingly. That’s certainly a good thing. This doesn’t yet exist in this form. […] And there are also many questions, and it’s indeed the case that the range is definitely very individual, which is why I generally like questionnaires for addressing special needs, because it facilitates ward rounds as well, since you can specifically address the issues.	Pneumologist, pulmonary rehabilitation (male, 18 years in patient care)
	Q4	And they all have completely different characters. There are some patients who do ask questions, and they get their information, but more than 50% of the patients listen to everything you say, then they leave and they feel like: Oh, actually I also wanted to ask this or that and how, so that doesn’t really help me now after all.	Pneumologist, pulmonary rehabilitation, (male, 18 years in patient care)
	Q5	When there is someone who is an expert on his disease, well I obviously know what kind of person they are just because of the treatment. Well, yes, I know whether it’s rather easy for them. […] I know whether they see the disease as a challenge. I know whether they have psychosocial circumstances, whether they panic or whether they’re afraid of their next asthma attack. I know whether they are usually in the self-help group. […] But with some people, well, you have to drag every word out of them. […] And then I’m like: Oh well, he won’t come regularly. I don’t even know, if he regularly takes his medicaments or how he takes them. Somehow, he’s just hopping from one physician to the next. So, in such difficult cases, I might use it and I would say: […] The outcome isn’t really okay. Now, we have to get together. What are your expectations? What do you wish for? To challenge them a little bit, I would say, and to tell them: Look, here’s the thing. We’re ready to do something, yes, we want to respond to your wishes. But then, we have to agree on a goal.	General practitioner (male)
	Q6	In order to really meet the patients where they actually stand. To have this written down as well, so that you can in fact see afterwards, after a year or maybe after one and a half years depending on their wishes in the first questionnaire / to realize this, to be able to really address their requests and to find out: Were we able to manage all of this? Or has the need simply changed as well?	Medical assistant, pneumological practice (female, 10 years in patient care)
	Q7	Well, I’d say you could basically use it as a quality check. So that the patient receives it at the beginning of his pulmonary rehabilitation but also again at the end of it. To simply compare what he has wished for, you know? What was basically important for him to know, to learn, and was it possible to implement that in the time?	Medical assistant, pneumological practice (female, 19 years in patient care)
	Q8	I would also like to see these questionnaires in disease management, for example, to identify patients who still need, let’s say, very specific information. So that we and also general practitioners feel like, oh wait, my patient seems to have quite a lot of questions left, I might introduce him to a pneumologist, or I’ll conduct a training program. The number of patients with respiratory diseases in the disease management program is the lowest compared to all management programs. And this already shows that there is indeed a supply gap, and the percentage of people who are also seen pneumologically is extremely low. And I can imagine that such a questionnaire could also be used in such a management program, […] so that you can see which patients can beneficially be presented by their general practitioner. Maybe in combination with the questionnaire to be able to immediately say, here, you can do something about that.	Pneumologist, pneumological practice (male, 35 years in patient care)
	Q9	Because I think it’s really important for a patient to know well about their disease. And it’s often the case that it’s quite a new situation for them, I would say it’s routine for the physician and for the assistants after all. And I think it’s not unusual that you forget to explain certain things or what seems normal for us, I suppose, what we know already, and the patient would actually like to get more information. So that these things just don’t get lost.	Medical assistant, pneumological practice (19 years in patient care)
*Implementation*			
Setting	Q10	Because it’s more specific and because I might feel like my pulmonologist is up to date in his special field, possibly in a different way than my general practitioner might be.	Patient (female, 53 years, 28 years since diagnosis)
	Q11	I would say there are few general practitioners who know so much about it that they are in the right position for it. I mean, I wouldn’t go to the dentist with a surgical issue either, for example.	Patient (female, 61 years, 31 years since diagnosis)
	Q12	Pneumologists would benefit more from such a survey, compared to general practitioners who have many other problems, diabetics and so on and so on, you know.	Pneumologist, pneumological practice (male, 30 years)
	Q13	He is the wrong contact person. I think, he is a difficult contact person for an asthmatic because he covers a completely different supply area. He lacks specific knowledge. […] And no offense, but therapies have completely changed over the past ten years. They have become more specific, and you can’t overlook all of that as a general practitioner. Well, and they often use therapies that should actually only be applied in case of an emergency, I would argue, be it Diophylline or oral cortisone, and some general practitioners in fact prescribe it even for a longer time and sometimes with side effects in long-term therapy.	Pneumologist, pulmonary rehabilitation (male, 18 years in patient care)
	Q14	Pneumologists know exactly what they have to do. I mean, that’s what they’re doing every day, right? General practitioners not so much, I mean, some education for general practitioners would be nice, wouldn’t it? My general practitioner has also often prescribed the wrong asthma inhaler to me. I told him about the exact side effects I was having, and he prescribed a different one. But it had the same components in it.	Patient (female, 65 years, 11 years since diagnosis)
	Q15	You said that they had been used in pulmonary rehabilitation clinics before. Well, in a rehab clinic, they have, I don’t know, twenty patients in their ward and they stay there for three weeks. This is a completely different setting obviously. If I want to use such a questionnaire there, I have basically three weeks to complete it during daily visits. I have twice as many patients here every day sometimes and I have to guide them through somehow, but there are new patients every day. […] I’d say we all want to do our best for the patients, the established pneumologists as well. The problem with such a questionnaire is that it often causes additional work and that the patients might think: “Oh, they can make more demands and the physician needs to have more than ten minutes for me now.” […] If an average routine patient starts thinking about it because of a questionnaire like this: “Oh, yes, I’d like to know what my medicament can also do and what it can’t do. Or what do I have to pay attention to?” We can’t manage that anymore.	Pneumologist, pneumological practice (male, 25 years in patient care)
	Q16	So about six minutes I have per patient.	Pneumologist, pneumological practice (female)
	Q17	Well, I have to be honest and say that with asthma, I can already see that the most important thing is actually the therapy. […] I think that medication is clearly in the focus of attention in order to treat the patient.	General practioner (female, 21 years in patient care)
	Q18	I can only emphasize that we do work a lot with the patients in our rehab. We educate a lot, we instruct them, and I can only support something like that, I think it’s just great. […] And as I said, the patients’need in the outpatient sector, they really are in need sometimes, […] the pneumologists just don’t have enough time for that.	Medical assistant, pulmonary rehabilitation (female, 11 years in patient care)
	Q19	This fits to the feeling I have sometimes, that the pneumologists have actually withdrawn from the outpatient program a bit more and that they do less simply because of the so-called factor of time pressure. But this is in fact the part that is most useful in the end. And like I said, well, an ambulant patient program, you know? Like I said, the pneumologist doesn’t have to do it himself, it can be delegated or at least a great part of it. And well, I do think that it’s their duty in a way in the outpatient sector. And we as a pulmonary rehabilitation clinic, we have these educational measures, training programs and so on, that’s a focus. But just think about how many times a patient comes here […] and how often do they see their pneumologist? Clearly, the focus on generating knowledge and practical handling is in the outpatient section, isn’t it? Especially in the repetition, especially in the starting situation, when it’s newly prescribed.	Pneumologist, pulmonary rehabilitation (male, 32 years in patient care)
	Q20	Actually, the time requirement naturally comes with the disease with these patients. You can’t just work it off in five minutes. So you should actually expect the physician to take enough time for it.	General practitioner (32 years in patient care)
	Q21	If we had this information before the training program, it would be a different setting. Then, you can prepare yourself a bit and say: ‘Oh gosh, quite a lot of people had these issues now.’ And you can really process it in the training. […] It would be a cause for thought for the patients, before the training as well, when they have the opportunity to ask their questions or to tell us what to do in advance. I think it would be really helpful in this case (Note: This refers to the DMPs).	Pneumologist, pneumological practice (male, 25 years in patient care)
	Q22	I think the best way to reach most people who are also sensitized for the topic and who would have time to fill it out is during pulmonary rehabilitation or at the lung specialist.	Patient (female, 53 years old, 18 years since diagnosis)
	Q23	So that they, yes that there is a forum with eight or ten people, three hours with a respiratory therapist, three hours with a physician and you are in a small group where you can open up, and important questions are in fact asked if they really burn on the heart, on the liver or wheresoever, on the lungs (Note: This refers to the DMPs).	Pneumologist, pneumological practice (male, 35 years in patient care)
	Q24	Well, let’s put it that way, such a questionnaire probably wouldn’t have a great influence on the course of pulmonary rehabilitation, because, like I said, many questions that are asked here are already covered by the standards, you know? Breathing techniques for example, as well. Or what you have to do in case of an acute asthma attack. These are exactly the parts that are dealt with in the asthma training program, precisely.	Pneumologist, pulmonary rehabilitation (male, 32 years in patient care)
	Q25	Well, yes and I think education is really, really important. What kind of disease is it? I can’t cure it or get rid of it. I can treat it symptomatically and I as a patient have to keep up. And you need to be reminded of that from the beginning onwards, again and again I would say, because everyone stops taking their medicaments as soon as they’re feeling better. Or they don’t go to their pneumologist regularly and they think: Well, it’s fine. Until it’s getting worse, and then you have to start all over again. You could actually avoid that.	Patient (female, 53 years, 28 years since diagnosis)
	Q26	I have only been to rehab, once. […] I don’t know if it meets the standard or if this is always the case no matter where you are. Or if that’s different in other clinics. But I know that we had a meeting with, let’s say, twenty or thirty people in a lecture room. I’m sure not everyone would have liked to talk about the questionnaire in front of the assembled company.	Patient (male, 58 years old, 8 years since diagnosis)
Time of first deployment	Q27	I wouldn’t use it after initial contact, there are too many open questions at this point, I would say it’s too specific and these questions address patients with a diagnosis who already have an established therapy and who know how it’s done. And I’d say the earliest point is after the second or third appointment after an allergy test, after a pulmonary function test and so on. The earliest time would be as soon as you say, I think we got the right adjustment now	Pneumlogist, pneumological practice (male, 35 years in patient care)
	Q28	I’d actually give it immediately at the initial consultation, so as soon as you get the diagnosis, because there are certain things that you might not be told right away like breathing techniques and so on. And then, you can give feedback to the physician and tell him where you still have needs. Things you might not have understood at once, because you don’t/ so, I’d say it shouldn’t be filled out immediately by the patient. I’d give it to the person who has just been diagnosed, as a kind of guidance.	Patient (female, 34 years, 23 years since diagnosis)
Frequency	Q29	So just that patients, for example, realize once again: Do I actually know about everything, or have I forgotten some bits and pieces yet? Or has anything changed, for example personal environment? That definitely makes sense.	Patient (male, 53 years, 31 years since asthma diagnosis)
	Q30	New patients who come to the practice for the first time, maybe. I think this could be quite interesting. So that you get some kind of overview. What do they know already or what questions do they have? And they could also be invited to the training as well. That would be my idea, at the beginning, at first contact. That you just staple it to the medical history. So, if they already have a diagnosis of asthma or something like that, I would do it with the anamnesis right away, to simply know about the present situation.	Medical assistant, pneumological practice (female, 17 years in patient care)
Responsible contact person	Q31	Well, I would mainly trust the physician, I’d say, at least at the beginning. This would be different once I feel a bit securer, for example now. Then, I can also talk to other people. […] But you feel very insecure, especially at the beginning.	Patient (female, 44 years, 8 years since diagnosis)
	Q32	I personally think that there is usually a good relationship of trust with the practice team. If they are trained, if they are able to provide helpful information, then I think it’s quite useful.	Patient (female, 61 years, 31 years since diagnosis)
	Q33	… that we employ pneumological assistants who question and examine patients especially in the disease management program as well. And this would be on a different level, it wouldn’t be patient and physician, which is often not a balanced conversation, but rather patient and pneumological assistant, and sometimes, […] different needs are expressed, something that wouldn’t necessarily happen in conversation with the physician.	Pneumologist, outpatient practice (male, 25 years in patient care)
	Q34	Many patients came to us and asked questions, especially the questions that are on the questionnaire, they asked me or us as medical assistants. I think it’s easier to ask us than to ask the physician, it’s less restrained. And it’s often the case that some questions come up while we take care of the patient, when we take an ECG or blood samples. And it’s always nice when they casually mention, oh, I have another question. Indeed. And the patients are very grateful when we provide them with information.	Medical assistant, general practice (female, 45 years in patient care)
	Q35	I try to put myself in my trainees’ place. But no, my trainee would also be able to answer every question, they’d know the answers.	Medical assistant, general practice (female, 45 years in patient care)
	Q36	It’s interdisciplinary after all. A physiotherapist must do that, they practice breathing techniques with them, our care department practices self-monitoring via peak-flow-meter with them, the handling of the asthma inhaler, the care section practices this. Of course, other occupational groups have to be included, as well. At least in the area of pulmonary rehabilitation. Right? That’s our goal after all.	Pneumologist, pulmonary rehabilitation (male, 32 years in patient care)
	Q37	Yes, but we as physician assistants, for example, we notice it again and again: The physician does ward rounds, or the assistant medical director, whoever, and they definitely tend to explain everything in their technical, medical jargon, and the patients are always like, they are merely nodding through, yes, got it, yes, and as soon as the physician is gone and they meet us again later, they’re asking us: “I didn’t really understand what the physician has just said.” Then, we often explain everything in more detail again so that the patient can also understand it, yes.	Physician assistant, pulmonary rehabilitation (female, 11 years in patient care)
Barriers	Q38	From my experience it’s instantly dismissed sometimes, with a certain hand gesture, you know? Like, no, it can’t be like that. And then you somehow seem to be the stupid one.	Patient (female, 64 years old, 10 years since diagnosis)
	Q39	What do you do when there is always the need for more consultation? But you know that you won’t have more than ten minutes the next time. I think that’s always difficult.	Pneumologist, outpatient practice (male, 25 years in patient care)
	Q40	I mean, the last question really kills any pneumologist in an outpatient practice. “Would you like your physician to make more time for you in case of special requests?” This is, well almost everyone will tick “yes” there or at least most of the patients will say “yes”. I mean, […] we regularly have four patients per hour in our practice. My colleagues regularly see six people already and additionally the acute cases and everything else in between. And we might have some people who would say that it’s already fulfilled. But otherwise, well, what do you do when you see that there is always the need for more consultation? But you know that you won’t have more than ten minutes the next time.	Pneumologist, outpatient practice (male, 25 years in patient care)
	Q41	Questionnaires usually end up in a drawer and aren’t really used in the daily routine.	General practitioner (male, 24 years in patient care)
	Q42	For example, when I ask one of my patients: “Do you need more information on the handling of the asthma inhaler?” And he says: “No, I know all that.” And then I have him demonstrate it and I see that he’s doing it all wrong, then there’s a significant bias in it.	Pneumologist, pulmonary rehabilitation (male, 18 years in patient care)
	Q43	“Yes, yes, I’ve been doing that for twenty years.” That’s the typical answer you get and then you dig deeper, and you tell them: “Oh, why don’t you demonstrate how you do it?” And then you notice some serious mistakes in the application and that the medicament can’t really get to where it’s supposed to go.	Physician assistant, pulmonary rehabilitation (female, 11 years in patient care)

Discussion	Q44	I’ve had it for thirty years and every now and then, I’ve had some crucial experiences in my patient career where I have actually been informed, after years, how to take the asthma medication, for example. Or what do to when I can’t breathe well. […] And I was like: Okay, you could have explained that to me five years earlier, I would have felt better faster probably. That’s why I certainly know what you’re writing about, when it comes to these questions now.	Patient (male, 53 years, 31 years since asthma diagnosis)
	Q45	So, it’s usually not the case that asthmatics complain about not having enough information or so. That’s indeed rather an exception. It’s more like we have to motivate asthmatics to come see us regularly, also when they are stable, that they still have to come see us once or twice a year and that they don’t just reorder their medication and that’s it.	Pneumologist, outpatient practice (male, 25 years in patient care)

#### Utility from clinicians’ point of view

**Overall impression** Most health professionals found NEAT useful, its implementation desirable, and perceived that the content of the questionnaire covered patients’ key health care needs (see verbatim quote Q1, Table 3). They found the items regarding information on drug effects (especially side effects and interactions), regarding information and training related to handling of drugs (especially correct use of asthma spray) and regarding exacerbations (practicing breathing techniques and more information on how to behave during an asthma attack) particularly useful.

Some health professionals found NEAT less useful, because they perceived its items as redundant to the content of DMPs and expressed that corresponding topics would be clarified there anyway. Some health professionals also explained that such health care needs were not an issue for many patients with asthma.

It would be desirable for several health professionals to include non-medical items on information about exercise, smoking cessation, and asthma at work, thus specifying the previous item on life circumstances. (i.e., “Would you like your physician to consider your personal circumstances to a larger extent in the treatment of your asthma?”).

**Physician-patient communication** Health professionals believed that the NEAT could positively influence physician-patient communication by facilitating the flow of information. In this context, they expressed that a more structured conversation using the NEAT could help to focus on the patient’s main concerns (see Q2–3), ensure that patients did not forget anything during the conversation (see Q4), and possibly save time. In addition, health professionals believed that patients would feel taken more seriously by using the NEAT in conversations and it could help to build trust (especially at the beginning of the physician-patient relationship). According to many participants, it would also be possible to better engage patients, especially those who were reserved, introverted or seem to show low adherence (see Q5). According to some health professionals, treatment could also be optimized through the use of the NEAT. Repeated use of the NEAT could serve as a progress or quality control in treatment (Which needs could already be met, which ones not yet?, see Q6-7). Patients with particularly high health care needs could especially benefit from this (see Q8). Health professionals frequently expressed that the tool could raise awareness of key asthma-related needs among patients (e.g., through increased confrontation with one’s own illness and through feedback to oneself), but also among physicians and health professionals in general (e.g., they might find out what needs exist in the first place and learn not to take certain knowledge for granted, see Q9).

#### Implementation

**Setting** Regarding its use in routine care (and outside the DMPs), patients tended to recommend more often using the NEAT in pneumological practices. Patients seemed more likely to perceive the pneumologist as a (confidential) contact person for their asthma rather than their GP and attribute more expertise to him or her (see Q10–Q11). Some GPs and pneumologists expressed a similar opinion, that is, that GPs had to treat a very wide range of diseases and thus they were less experienced in treating asthma compared to pneumologists (see Q12). Some pneumologists (in outpatient and pulmonary rehabilitation setting) also expressed concerns regarding a lack of expertise among GPs (see Q13). The implementation of the NEAT in primary care was only recommended by patients, pneumologists and pulmonary rehabilitation staff in case patients do not have access to a pneumologist (e.g., in rural areas) or prefer to be treated only by their GP. However, a common suggestion was that the use of the NEAT could help raise awareness especially among GPs that patients with asthma need more information and training (see Q14).

Nevertheless, pneumologists and GPs were generally rather skeptical regarding the implementation of the NEAT in outpatient practice (outside DMPs), as economic pressures and very high patient volumes would make it impossible to consider unmet needs (see Q15). In particular in pneumological practice tightly structured practice procedures were perceived not to allow for time flexibility (pneumologists reported being able to schedule 6–10 min per patient, see Q15–Q16). Therefore, physicians had to set certain priorities in treatment, e.g., medication was more urgent than the use of NEAT tool (see Q17).

However, pulmonary rehabilitation staff perceived clear deficits with regard to patient education and training in outpatient settings (Q18). Outpatient physicians treat patients much more frequently and over many years, and therefore patient education should not only be outsourced to pulmonary rehabilitation in their view (Q19). Some outpatient physicians shared this view and emphasized the need to take time for patient education (Q20). Deployment of the NEAT in primary care could also be done to identify patients who should additionally contact a pneumologist based on their health care needs (e.g., to get more specialized information, see Q8). Furthermore, it would be useful to use the NEAT in primary and secondary care to identify patients in need of education and training (see Q8).

Overall, it appears that both patients and health professionals tended to recommend the implementation of the NEAT in the context of patient education, i.e., either during pulmonary rehabilitation or before or during DMPs (see Q21–Q22). The main reason for this suggestion was that in those settings sufficient time (of both patients and clinicians, see Q15, Q22) is perceived to be given and that there is a match between the inquired needs and the availability of immediate measures to meet them through educational opportunities (e.g., exercising breathing techniques or handling the asthma spray, see Q23).

The only criticism expressed by pulmonary rehabilitation staff was that the administration of the NEAT in pulmonary rehabilitation settings could be redundant, i.e., that the mentioned topics will anyway be addressed during the training and during ward rounds (see Q24). According to pulmonary rehabilitation staff, lack of patient education was more of a problem in outpatient medical care than in pulmonary rehabilitation clinics (see Q19). Patients expressed concern that a first utilization during pulmonary rehabilitation might be too late or that patients not participating in any pulmonary rehabilitation would not have access to the questionnaire. They considered the use of the NEAT as part of patient education to be important, especially at the beginning of the disease (see Q25).

Health professionals, as well as many patients, describe group conversations during pulmonary rehabilitation or DMP training as an appropriate format for meeting unmet health care needs. However, some patients felt it was the wrong setting to meet their individual needs (see Q26) and preferred a two-way conversation with the attending physician (e.g., in pulmonary rehabilitation during ward rounds, and in DMPs in the course of medical check-up).

For a complete overview of advantages and disadvantages of different settings, see Table 4.

**Table 4:** Advantages and disadvantages of different settings, patients’ and clinicians’ view

Setting		Patients	Health professionals
General practice	Yes	To improve patient education	
		Some patients do not have a pneumologist (not available or wanted)	
		For awareness among GPs of patients’ needs (What does the patient really need?)	
		To identify patients who should be seen by a pneumologist	
			Most frequent and close patient contact
			Time should be taken to clarify unmet needs
	No	Missing expertise of GPs	
		No time in everyday practice	
		Lack of willingness of GPs	
Pneumological practice	Yes	High expertise of pneumologists	
			Frequent patient contact
			More effective conversation management
			Inquire about need/optimize therapy
			To improve patient education
			To control the success of the treatment
	No	No time in everyday practice (structured practice procedures, High patient volume, economic pressure)	
		Some patients do not have a pneumologist (not available or wanted)	
Disease Management Program (DMP)	Yes	Identification and evaluation of health care needs (Which needs exist before, which could be met afterwards?)	
		Provides sufficient time (staff and patients have more time, group meetings save time)	
		During training (group discussion)	
		Short two-way conversation with physician	
	No		Too structured (no room and time for flexibility)
			Redundant (mentioned needs are addressed anyway)
Pulmonary rehabilitation	Yes	Provides content frame (education and practice, pulmonary rehabilitation staff get more information about patients)	
		Provides time frame (staff and patients have more time)	
		Identification and evaluation of health care needs (Which needs exist before, which could be met afterwards?)	
		Especially if diagnosis is recent	
		High expertise of training staff	
	No	Usually too late in medical history	
		No willingness among staff	
			Redundant (mentioned needs are addressed anyway)

**Time of first deployment** Patients and health professionals seemed to consider it more useful to use the NEAT some time after the initial diagnosis had been established. Health professionals recommended that the questionnaire should only be used after the patient has been adjusted to medication (otherwise, unmet needs may also be related to the fact that medications were not yet working, see Q27). They proposed the first medical check-up appointment (usually two to three months after the diagnosis) or an even later appointment for the first utilization of the NEAT. Like patients, they consider it necessary that initial education has already taken place. Patients would then already have some basic knowledge about their asthma and have had adequate time to emotionally process the diagnosis.

Patients were more likely than health professionals to see the benefits of using the NEAT immediately after diagnosis or at the time of diagnosis (see Q28). Such an early deployment could help to deal with the disease (e.g., addressing insecurities), could represent a first step in the education process and might support a prompt start of a (patient-centered) treatment strategy.

**Frequency** When used in an outpatient setting, patients said it may be useful to complete the NEAT routinely in order to be able to identify changes related to their health care needs (see Q29), to check their actual knowledge about their own disease, to correct adverse disease behavior and to be able to obtain additional patient education. Especially the latter was particularly important to some patients, as they reported to feel poorly informed about their asthma and its treatment even years after diagnosis. If the NEAT was to be used regularly in the outpatient setting, health professionals suggest its use once a year. However, they considered situational use to be easier to implement. For individuals with poor outcomes, such as low asthma control, poor drug adjustment, low compliance or exacerbations, asking for needs could help identify causes. Patients also considered a situation-specific use of the questionnaire as possible, especially in the case of poor outcomes. However, they advised against using the NEAT directly after exacerbations. They perceived this as too late in the course of the disease (as it should ideally take place before an exacerbation). Furthermore, they expressed the concern that the exhaustion after an asthma attack is perceived to be too great to deal with the contents of the questionnaire immediately afterwards and they would wish for more support in such a situation than just using a questionnaire. Another situation-specific deployment option that both parties saw would be to use the questionnaire only for patients with recently diagnosed asthma (see also *time of first deployment*) and/or patients new to the practice (see Q30). In this regard, some patients suggested using the NEAT repeatedly at the beginning of treatment until needs are met.

During pulmonary rehabilitation, patients and health professionals suggested using the NEAT for (patient-centered) therapy planning at the beginning (e.g., at admission), but also for evaluation rather at the end of the stay (preferably before the final ward round to be able to discuss deficits if necessary). In some circumstances, deployment at the end of pulmonary rehabilitation could also work with the intention to provide information to the outpatient pneumologist. Possibly, the NEAT could also be deployed on admission and after half the stay to still have the opportunity to meet unaddressed health care needs during pulmonary rehabilitation.

In the context of DMPs, health professionals suggested that the NEAT could be distributed (a) only one time, before the start of the patient training (e.g., together with the invitation to the training), patients would complete the questionnaire at home and submit it with the registration; or (b) twice, before and after patient training for evaluation. Patients found the routine use of the NEAT within the DMPs and supplementary to physiological measures (like pulmonary function measurement) useful, e.g., once per quarter.

For a complete overview of time of initial use and frequency of use, see Table 5.

**Table 5:** Time of initial use and frequency of use in different settings, patients’ and clinicians’ view

Setting	Initial use		Frequency	
	Patients	Health professionals	Patients	Health professionals
General practice/ Pneumological practice	**(A)** **Immediately after diagnosis**		**(A) Routinely**	
	• Initial overview of needs for patients and health professionals		• Capture changes	• Once a year
	• To deal with the disease		• Check knowledge level	
	• Prompt start of treatment		• Correct adverse disease behavior	
			• To get routine in dealing with asthma	
	**(B) From the first control appointment**		**(B) Situation specific**	
	• Time to process diagnosis		• Why poor outcomes?	
		• When patient has already received initial information	• For low asthma control	
			• For poor drug regimen and medication changes	
			• Not directly after exacerbations (too late, too exhausted, questionnaire then insufficient)	• For exacerbations
				• For low compliance
Disease Management Program (DMP)		• Before DMP (as preparation, identification of training needs and evaluation)	**(A) Routinely: once per quarter**	
			• Supplementary to physiological measures	
			• Feedback for physician	
			• Short clarification through physician	
				**(B) One time: before DMP patient education**
				**(C) Twice: before and after DMP patient education**
				• For evaluation: Were unmet needs met?
Pulmonary rehabilitation	**(A) On admission**		**(A) Twice: On** **admission and** **discharge**	
	• Need-oriented therapy planning / for more focused education and practice		• For evaluation: Were unmet needs met?	
	• To consider current state of knowledge	• In course of the initial formalities	• As feedback for patients and pulmonary rehabilitation staff	• As information for the outpatient sector
	• To inform pulmonary rehabilitation staff about needs			• To discuss unmet needs at final ward round
				**(B) Twice: On** **admission and** **after half the stay**
				• Possibility to still respond to needs
			**(C) One time: at admission**	

**Health professionals responsible for discussing/addressing needs** Most patients and health professionals felt that the physician, as an important contact person and confidant in the physician-patient-consultation, should discuss and address identified needs with patients (see Q31). This was found to apply to pharmacological topics such as medication intake, side effects and interactions. They agreed that other unmet needs could also be discussed and addressed with the help of medical assistants (e.g., correct handling of asthma spray or practicing breathing techniques), because physicians lack the time and medical assistants were perceived to have the required expertise. In addition, medical assistants and physicians as well as some patients expressed that patients often show a high level of trust in medical assistants (see Q32). Patients are less restraint during personal contact, as communication is perceived to be at eye level, but also easier to understand (see Q33–34). Some medical assistants (in pneumological and general practice) also had the confidence to address all aspects of the NEAT with patients independently (see Q35).

In pulmonary rehabilitation clinics, patient education is carried out by respiratory therapists, physiotherapists, or physician assistants, among others. A division, e.g., into aspects that are discussed during the ward round or in the physician-patient consultation and those that are clarified by other therapists during the patient training, already takes place and could be done in a similar way for the topics covered by the NEAT (see Q36). Similar to the outpatient setting, non-physician staff described that it can be helpful when very complex topics, such as those explained by the physician during ward rounds, are later repeated in simpler terms by non-physician staff (see Q37).

**Barriers** Barriers expressed by patients relate primarily to the interpersonal level in physician-patient contact: They were concerned that patients may not insist that their health care needs will be met (e.g., because they are worried about the physician’s reaction). In addition, some patients may be unwilling to provide information about very private aspects of their personal circumstances (e.g., psychosocial aspects). They also supposed an unwillingness on the part of physicians to address patients’ needs expressed in the NEAT and saw a risk that treating physicians will not take patients with their unmet needs sufficiently seriously (see Q38).

Certainly, the most important barrier that health professionals (but to a lesser extent patients) in outpatient settings expressed was the time commitment that would be required in using the questionnaire (see also *setting*). Both pneumologists and GPs often did not see time to meet unaddressed patient needs in their day-to-day practice (see Q39) e.g., due to fixed practice structures and no monetary compensation when using the NEAT. Furthermore, they are concerned that the questionnaire could elicit (additional) needs that the patients were not aware of before. In addition, the NEAT implies the promise to be able to address these needs in everyday practice (see Q40). Particularly the item “Would you like your physician to make more time for you in case of special requests?” was perceived to carry the risk of creating expectations towards the practitioner that cannot fully be met (see Q40). Nevertheless, there were also GPs and pneumologists who holded the opinion that physicians should take time for the patients’ needs. In this way, they would be more likely to establish a relationship of trust and cover psychosocial dimensions of treatment. However, even apart from time aspects, successful and long-term implementation of NEAT into existing practice processes was often perceived as difficult (mainly because questionnaires are forgotten in everyday practice, see Q41).

Barriers on the side of patients that were perceived by health professionals related to various patient characteristics. In this context, a potential lack of motivation of certain patient groups was often mentioned: E.g., patients of younger age, with low asthma severity, seasonal asthma, or even generally low compliance were generally perceived to express less health care needs and might be rather unmotivated to engage intensively with NEAT. In addition, health professionals were concerned that biased patient self-assessment may lead to only partially valid response behavior when completing NEAT. For example, patients that express to be convinced to handle their asthma inhaler correctly, often fail to use it correctly from the physicians’ point of view (see Q42-43). Furthermore, health professionals (but also patients) mentioned that for certain patient groups such as people with dyslexia or cognitive impairment (for the latter a version in simple language would be necessary) or people with a migration background (the translation of the NEAT into other languages would be useful) are not taken into account so far.

## Discussion

The majority of health professionals perceived the NEAT to be useful, its implementation as desirable, and believed that NEAT could positively influence physician-patient communication, although they were likewise skeptical with regard to its routine use (see below). Consistently, health professionals rated NEAT as very useful for pulmonary rehabilitation as well as for primary and pulmonary care based on a standardized questionnaire. Furthermore, it seemed desirable for several health professionals to include items that cover non-medical issues (e.g., information on exercise, smoking cessation, and asthma in the workplace). The reason why these items have not been included is that in a previous study [3] (a) patients found certain topics significantly more important than others and (b) statistical analyses ultimately led to the present four subscales with a total of 13 items. Therefore, we can assume that NEAT represents key patient needs, which may not fully correspond to what health professionals define as needs (i.e., “normative needs”). In the context of PROMs the term *need* should be understood as a perceived health care need that is felt by a given patient and remains unmet [21, 22] It should also be mentioned that the structure of the questionnaire (e.g., the response format and length) were developed together with patients and therefore primarily correspond to their preferences [3].

Above all, health professionals and some patients described time pressure as a key barrier regarding the implementation of NEAT especially in pneumological practices, but also in primary care. Although physicians sometimes felt that the NEAT could make procedures more efficient, there was often the concern that using NEAT would add to existing time pressures. In this context, other research findings also suggest that the use of PROMs could be very time consuming [23], while still recognizing that their results could be used to prioritize problems and increase efficiency [24, 25]. Therefore, one of the recommendations in the scientific literature (similar to the recommendation of some physicians in the present study) is to develop questionnaires that are as short as possible [23]. However, there is also evidence that increased time spent on patient visits does not need to occur as a consequence of using PROMs [26]. Regarding NEAT, outpatient physicians were concerned that the questionnaire could elicit needs that patients had not been previously aware of and that some needs (e.g., “Would you like your physician to make more time for you in case of special requests?”) could not be met in everyday practice. Overall, pneumologists and GPs therefore tended to be critical of routine use of NEAT in the outpatient setting.

In contrast, implementation of the NEAT in the context of DMPs (e.g., as preparation for and follow-up in the context of patient education) was strongly recommended by health professionals and patients alike. According to both parties, DMPs offer the possibility to answer questions, to perform exercises, and to check whether specific unmet health care needs exist. A patient enrolled in a DMP could receive the questionnaire in a standardized way as preparation. This would make the patient aware of educational topics in advance. The unmet health care needs could then partly be met with assistance of medical assistants (e.g., regarding the correct use of the asthma spray) and partly by the treating physicians (e.g., regarding side effects). In this context, other studies also suggest that in clinical practices with multidisciplinary teams, the various skills available could be used to address patients’ particular issues [27] and could reduce the time challenge for the physician [25]. Equally supported by both patients and health professionals is the implementation of the NEAT in pulmonary rehabilitation. In this setting, sufficient time is scheduled for training and information and thus the implementation of the NEAT would make sense both in terms of time and its content. Utilization at the beginning (therapy planning) and at the end of pulmonary rehabilitation (evaluation) would be conceivable.

It is important to consider that, similar to the pulmonary rehabilitation staff and unlike the majority of pneumologists and GPs, patients recommended implementation of the NEAT in routine care in the outpatient setting and, in particular, in pneumological practices (primarily due to the high level of expertise). One reason why outpatient physicians are less likely than patients to see the possibility (and importance) of using NEAT in outpatient care might be that patients and health professionals expressed partly contradictory assessments of the quality of education, information transfer, and patient health care needs. While some patients reported long disease careers with a lack of information flow in outpatient setting (see Q44), outpatient physicians and medical assistants tended to classify patients with asthma as less motivated and with little need for information (especially compared to patients with COPD, see Q45). This appears to be in line with the results of a previous study, which found that patients perceive and evaluate aspects of treatment (e.g., quality of communication or time spent on patient education) differently than the treating physicians [2]. It could also be related to the fact that outpatient physicians (especially GPs) in our study reported that they treat many patients with mild or only seasonal symptoms who appear to have a low disease burden and tend to have low compliance. Pulmonary rehabilitation staff, by contrast, was more likely to treat patients who experience asthma-related impairment, i.e., people who may have more unmet health care needs. This notion is supported by prior research: in a previous study we found a mean of 5.73 NEAT needs at admission to inpatient pulmonary rehabilitation [6] compared to 3.37 unmet needs in the present sample. Pulmonary rehabilitation staff was also more likely to describe patients as motivated. This could explain why they suggested using the NEAT not only during pulmonary rehabilitation, but also in the outpatient setting, in order to be able to work better preventively and to avoid exacerbations and high disease burden.

### Implications for policy, practice, and further research

The NEAT is a validated instrument [5] that has already been used in clinical practice, i.e., in three pulmonary rehabilitation clinics [6], and has now been evaluated together with patients and health professionals. Since the results indicate that the NEAT could be used primarily in the context of patient education, the NEAT should additionally be used and evaluated in the DMPs context. While some patients and some members of the pulmonary rehabilitation staff have already gained experience with the NEAT in the pulmonary rehabilitation setting during a previous study [6] (and were able to share this in the current interview study), this would also be useful in DMP setting with patients, physicians, and medical assistants.

While PROMs are commonly used as established tools with robust standards in research, their utility for health care decisions is generally poorly understood [28]. A recent Cochrane review indicates that the use of PROMs in clinical practice is likely to result in moderate improvements in communication between patients and health professionals as well as in diagnosis and disease control, and in small improvements in quality of life [29]. In order to be able to make similar statements regarding the NEAT, the questionnaire could be tested again in clinical practice in the form of an RCT (e.g., regarding asthma control or asthma-related quality of life), similar to the studies included in the review. Although quasi-experimental studies are also recommended to evaluate the effect of using PROMs [30–32] (as we have already done with respect to NEAT [6]), an RCT would minimize bias and confounding through randomization [32].

Our study also highlights the need to make the NEAT accessible to all patients with asthma. Therefore, it should be translated into additional languages (e.g., Turkish or Russian) and could be made available in simple language or for people with visual impairments.

In addition, our study indicates that there may be little time for patient-centered care based on formal tools to assess health care needs in outpatient practices. However, structural shared decision making in the outpatient setting appears to significantly improve adherence to asthma pharmacotherapy and clinical outcomes (e.g., asthma control and lung function) [33, 34]. More research in this area would be important. For example, regarding the question of what incentives would need to be provided to physicians to give more priority to person-centered care, e.g., through revised payment models such as incentive payments for reaching quality targets [35–37]. Thus, overall, one consideration should be that patient education time should be (better) compensated in the outpatient setting.

### Strengths and limitations

A particular strength of the study is that we were able to interview patients with asthma, as well as all health professionals involved in their care in various settings. Our approach was thus patient-centered, but also multi-perspective. Although only one coder performed the full qualitative analysis, two additional analysts contributed to the analytical process and could increase replicability, traceability and discriminatory power.

However, it should be taken into account that we may not have been able to represent the full range of possible views. The patient sample tended to have few unmet health care needs as measured by the NEAT (3.37 out of 13) and reported a high degree of asthma control (8.74 out of 10, measured by a question of the Brief IPQ). Furthermore, patients were more likely to report being currently satisfied with their health care, often because they had initiated changes themselves (e.g., a change of physician). Furthermore, patients were predominantly in the age group between 45 and 64 (which corresponds to the age group with the highest prevalence in Germany [38]) and had asthma for an average of more than 20 years. However, a previous study on NEAT [4] showed that patients at younger age (> 45) and who had recently received their diagnosis had more unmet needs. A more diverse recruitment strategy should be adopted in a future study. Nevertheless, the advantage of our sample was that several patients (eight of 19 participants) had already completed the NEAT on admission and discharge during their stay in pulmonary rehabilitation and as part of a previous study [6]. They were thus able to provide reliable feedback on how helpful the NEAT was for them in the above setting.

Similar to patients, it remains unclear whether we were able to represent the full range of possible view among health professionals. For example, there may be a bias that more motivated health professionals participated, or those who tend to be positive about person-centered care or NEAT (some of the health professionals already participated in another NEAT study). Thus, we may not have been able to include some critical perspectives and interview hard-to-reach people. In addition, because we interviewed so many different occupational groups, we were only able to interview a few participants per occupational group. While we continued data collection until the point where we felt that no new themes have emerged (i.e. thematic saturation) across all interviews, we cannot rule out that we have terminated our data collection too early in some occupational groups.

## Conclusion

Both patients and health professionals believe that the NEAT could positively influence the physician-patient communication and consider the use of the NEAT feasible and useful, in particular in educational programs. Although patients would prefer routine use of the NEAT in pneumological practice, health professionals currently see little opportunity to do so due to time constraints. Beyond the findings on the NEAT, our study provides preliminary evidence that there appears to be little scope for person-centered care in current outpatient care for patients with asthma in Germany.

## Supplementary Information

**Additional file 1.** Interview Guide: Patients

**Additional file 2.** Interview Guide: Health professionals

**Additional file 3.** COREQ (COnsolidated criteria for REporting Qualitative research) Checklist

## Data Availability

The datasets analyzed during the current study are not publicly available to protect personal data of the participants, but are available from the corresponding author on reasonable request.

## Abbreviations

COPD: Chronic obstructive pulmonary disease
DMP: Disease management programs
GP: General practioner
IPQ: (Brief) Illness Perception Questionnaire
NEAT: Patient Needs in Asthma Treatment questionnaire
PROM: Patient reported outcome measure

## Footnotes

1 In German heath care, medical assistants assist physicians with their daily administrative tasks and some basic clinical tasks. They usually work in outpatient (and sometimes in inpatient) care in close contact with patients.

2 Additional qualification in which certain activities delegated by the physician can be taken over. In the participating pulmonary rehabilitation clinic, this included, with regard to patient contact, the conducting of certain training courses and consultation hours as well as participation in the ward rounds.

3 In Germany, Disease Management Programs (DMPs) for asthma have been offered by the statutory health insurance funds in cooperation with physicians (pneumologists and GPs) since 2005. In addition to regular check-ups and counseling, these DMPs also include extensive knowledge transfer and promotion of self-management through appropriate training programs [39, 40].
