# Supplementary material for: Implementing the Patient Needs in Asthma Treatment (NEAT) questionnaire in routine care: a qualitative study among patients and health professionals
Source: BMC Pulm Med. 2023 Jan 17;23:21. doi: 10.1186/s12890-022-02293-4 (PMC9843883; doi:10.1186/s12890-022-02293-4)
Supplement: Supplementary file 1 — Additional file 1. Interview Guide: Patients [file 12890_2022_2293_MOESM1_ESM.docx]

**Interview Guide: Patients**

| A) Interviewer introduction (pulmonary health services researcher)  B) Study aim and information  "I am glad that you take the time to participate in our study.  I would like to explain a few things first and then start the interview, if you agree.  As announced, I would like to review a questionnaire with you about patient needs in asthma care. I am interested in whether and how you could imagine that the questionnaire could be used in the treatment of patients with asthma to better address patient needs in health care.  I want you to know that there are no right or wrong answers. Every answer helps us, and it is only about your personal opinion.  If you agree, I will record the conversation to make sure that important information is not lost. However, you can tell me to turn off the device at any time.  The interview will take about half an hour and it would be great if you have the questionnaire in front of you.  Before we begin, are there any things that are still unclear, or do you have any questions?  ...Then we will start the interview now." |
| --- |

C) Interview questions

| 1. Perhaps you could start by telling me how satisfied you are with your current asthma treatment and whether you have any particular wishes or expectations regarding your treatment? |
| --- |
| 2. What is your overall impression of the questionnaire?  A) What do you like about it?  B) Which questions do you find less appropriate?  C) Is any important content missing? |
| 3. Do you think that the questionnaire or certain questions are relevant for your treatment?  A) If yes, which questions do you find particularly relevant and why?  B) If no, which questions do you find not or less relevant and why? |
| 4. *If only 3B applies:*  Even if you think that the questionnaire is not particularly relevant to you personally, I would still ask you to answer the following questions keeping in mind that the tool might be helpful to other patients.  *If 2A (and 2B) applies, start right here:*  Next, I am interested in how exactly you would like to complete and discuss the questionnaire in your treatment.  There are no right or wrong answers, we are mainly interested in your ideas as a patient! |
| *Only ask questions 5.1 to 5.6 if the respondent has not yet addressed them independently.* |
| 5.1 Where should the questionnaire be applied, i.e., in which medical institution?  (*If no answer is provided: e.g., at your general practitioner, pneumologist, during rehabilitation, etc.?)*  Are there any other medical settings that would make sense from your point of view?  A) What would be the advantages and disadvantages of each setting?  *(Ask for advantages and disadvantages of all three settings.)*  B) Which setting would be best for you?  *(Ask B only if not already clear through interview.)* |
| 5.2 When, i.e., at what point in asthma treatment, should the questionnaire be used?  *(If no answer is provided: e.g., soon after diagnosis or rather a little later?)*  A) Should the questionnaire be used regularly, i.e., at every medical appointment?  B) Or only in certain situations, e.g., if your asthma worsens? |
| 5.3 How or where would you prefer to complete the questionnaire?  *(If no answer is provided: e.g., in a personal interview or by self-report in the waiting room or from home?)*  A) Could you explain your answer, please?  B) If in a face-to-face interview: Who should assist you in completing the questionnaire? |
| 5.4 In which form would you prefer to complete the questionnaire?  *(If no answer is provided: e.g., by tablet/PC or in paper form?)*  A) Could you explain your answer, please? |
| 5.5 Would you like someone to discuss the results with you?  A) If yes, why and how?  B) If no, why not? |
| *If question 5.5 is answered with "yes":*  5.6 With whom would you most like to discuss the results of the questionnaire?  *(If no answer is provided: e.g., with your physician or with a medical assistant?)*  A) Could you explain your answer, please?  B) If you were to discuss the results with your physician, would you have any concerns about it?  *(Note: B targets whether patients would fear negative consequences to their treatment or answer with social desirability due to an offended physician).* |
| 6. In your opinion, to what extent could the individual needs that are asked about in the questionnaire be better met by treatment?  A) What might be missing in current care? |
| 7. Did reading the questionnaire make you aware of any other aspects you would like to see in your asthma treatment?  A) What exactly would that be?  B) Do you already have an idea of what you could do better meet these needs?  *(If no answer is provided: e.g., get more information, talk to your physician, etc.)*  *(Note: A aims at whether NEAT could potentially raise awareness or promote disease perception. B targets whether NEAT could potentially lead to behavior change.)* |
| 8. Is there anything else you would like to tell me or that still seems important to you? |

**Thank you very much for the interview.**
