# Supplementary material for: Implementing the Patient Needs in Asthma Treatment (NEAT) questionnaire in routine care: a qualitative study among patients and health professionals
Source: BMC Pulm Med. 2023 Jan 17;23:21. doi: 10.1186/s12890-022-02293-4 (PMC9843883; doi:10.1186/s12890-022-02293-4)
Supplement: Supplementary file 2 — Additional file 2. Interview Guide: Health professionals [file 12890_2022_2293_MOESM2_ESM.docx]

**Interview Guide: Health professionals**

| A) Interviewer introduction (pulmonary health services researcher)  B) Study aim and information  "I am glad that you take the time to participate in our study.  I would like to explain a few things first and then start the interview, if you agree.  As announced, I would like to review a questionnaire with you about patient needs in asthma care. The questionnaire was developed together with patients, scientifically tested and already used in three rehabilitation clinics.  I am now interested in whether and how you could imagine that the questionnaire could be used in the treatment of patients with asthma to better address patient needs in health care.  I want you to know that there are no right or wrong answers. Every answer helps us and I am interested in your opinion as a subject matter expert.  If you agree, I will record the conversation to make sure that important information is not lost. However, you can tell me to turn off the device at any time.  The interview will take about half an hour and it would be great if you could have the questionnaire in front of you.  Before we begin, are there any things that are still unclear, or do you have any questions?  ...Then we'll start the interview now." |
| --- |

C) Interview questions

| 1. Could you start by briefly telling me how you work with patients with asthma in your day-to-day work? |
| --- |
| 2. Do you feel that patients with asthma often tell you about unmet needs regarding their treatment? |
| 3. What is your overall impression of the questionnaire?  A) What do you like about it?  B) Which questions do you find less appropriate?  C) Is important content missing? |
| 4. Do you think that the questionnaire or certain questions are relevant for the treatment of patients with asthma?  A) If yes, which questions do you find particularly relevant and why?  B) If no, which questions do you find not or less relevant and why? |
| 5. What do you think about the use of the questionnaire in your daily work?  *(If not understandable, add the following: e.g., easy, complicated, time-consuming, what barriers could exist?)*  A) Would the use of the questionnaire be possible in your everyday work?  B) Why not? What is currently missing? Which conditions would have to be fulfilled?  C) What about time aspects? |
| 6*. If 5A is answered with "no":*  Even if you cannot currently imagine using the questionnaire in your own everyday work, I would still like to ask you some questions about other possible uses.  *Otherwise, start directly here (if 5A is answered with "yes"):*  Next, I am interested in how exactly you would find it most useful to use the questionnaire in health care practice. This does not necessarily have to be your own everyday work. |
| *Only ask questions 7.1 to 7.3 if the respondent has not yet addressed them independently.* |
| 7.1 Where should the questionnaire be applied, i.e., in which medical institution?  (*If no answer is provided: e.g., at general practice, pneumological practice, during rehabilitation, etc.?)*  Are there any other medical settings that would make sense from your point of view?  A) What would be the advantages and disadvantages of each?  *(Ask for advantages and disadvantages of all three settings.)*  B) Which setting would be best for you?  *(Ask B only if not already clear through interview.)* |
| 7.2 Thinking now about your everyday rehabilitation/practice life: How, when and where could the questionnaire be applied?  *(If no answer is provided: Face-to-face or by self-report, in the waiting room or from home, with tablet or in paper form?)*  Could you explain your answer, please?  A) Who should discuss the results with the patients? |
| 7.3 When, i.e. at what point in asthma treatment, should the questionnaire be used?  *(If no answer is provided: e.g., soon after diagnosis or rather a little later?)*  A) Should the questionnaire be used regularly, i.e. at every medical appointment?  B) Or only in certain situations, e.g. if a patient’s asthma worsens? |
| 8. Could you suggest interventions that could be used to meet certain needs?  A) Are there needs for which no intervention would be effective? |
| 9. Are you concerned that your performance may be evaluated by the questionnaire?  A) Do you have any other concerns about the use of the questionnaire? |
| 10. In conclusion: Would the use of the questionnaire be desirable for you?  A) If yes, why?  B) If no, why not? |

| 11. I would now ask you to rate the following statements: | | | | |
| --- | --- | --- | --- | --- |
| I find the questionnaire very useful for.... | Do not agree at all | Do not agree | Agree | Fully agree |
| …general practice |  |  |  |  |
| …pneumological practice |  |  |  |  |
| …pneumological rehabilitation |  |  |  |  |

| 11. Do you have any other comments or did you notice anything else that you would like to share with me? |
| --- |

D) Demographic information

| 1. Type of profession | ______________________________________________ |
| --- | --- |
| 2. Professional experience in patient care | __________ years or since year: __ __ __ __ |

**Thank you very much for the interview.**
